# Evaluation of Laboratory Predictors for In-Hospital Mortality in Infective Endocarditis and Negative Blood Culture Pattern Characteristics

**DOI:** 10.3390/pathogens10050551

**Published:** 2021-05-02

**Authors:** Ana-Maria Buburuz, Antoniu Petris, Irina Iuliana Costache, Igor Jelihovschi, Catalina Arsenescu-Georgescu, Luminita Smaranda Iancu

**Affiliations:** 1 Department of Cardiology, “Grigore T. Popa” University of Medicine and Pharmacy, 6 University Street, 700115 Iasi, Romania; antoniu.petris@yahoo.ro (A.P.); ii.costache@yahoo.com (I.I.C.); catalina.arsenescu@gmail.com (C.A.-G.); 2 Department of Microbiology, “Grigore T. Popa” University of Medicine and Pharmacy, 6 University Street, 700115 Iasi, Romania; jelihovsky@yahoo.com (I.J.); luminitasmaranda@yahoo.com (L.S.I.)

**Keywords:** infective endocarditis, blood culture, prognostic, laboratory tests

## Abstract

Objective: This study aimed to identify possible differences between blood culture-negative and blood culture-positive groups of infective endocarditis (IE), and explore the associations between biological parameters and in-hospital mortality. Methods: This was a retrospective study of patients hospitalized for IE between 2007 and 2017. Epidemiological, clinical and paraclinical characteristics, by blood culture-negative and positive groups, were collected. The best predictors of in-hospital mortality based on the receiver-operating characteristic (ROC) analysis and AUC (area under the curve) results were identified. Results: A total of 126 IE patients were included, 54% with negative blood cultures at admission. Overall, the in-hospital mortality was 28.6%, higher in the blood culture-negative than positive group (17.5% vs. 11.1%, *p* = 0.207). A significant increase in the Model for End-Stage Liver Disease Excluding International Normalized Ratio (MELD-XI) score was observed in the blood culture-negative group (*p* = 0.004), but no baseline characteristics differed between the groups. The best laboratory predictors of in-hospital death in the total study group were the neutrophil count (AUC = 0.824), white blood cell count (AUC = 0.724) and MELD-XI score (AUC = 0.700). Conclusion: Classic laboratory parameters, such as the white blood cell count and neutrophil count, were associated with in-hospital mortality in infective endocarditis. In addition, MELD-XI was a good predictor of in-hospital death.

## 1. Introduction

Infective endocarditis (IE) has a relatively low incidence, but mortality remains high (15–30%) and has changed little in recent decades, despite new diagnostic methods and treatment options [1]. An epidemiological shift has occurred, changing it from a condition affecting young patients with rheumatic heart disease to one mostly affecting older patients, with degenerative valve disease or associated with healthcare interventions. In recent years, a microbiological change has occurred, from etiological agents such as viridans streptococci to more virulent ones, such as *Staphylococcus aureus* [2].

Infective endocarditis has many variable forms of presentation, from the classical association between fever and a new-onset cardiac murmur, to unspecific clinical aspects, such as embolic stroke, heart failure and absence of fever. The diagnosis of IE remains a challenge, and a combination of clinical, microbiological and echocardiographic findings forms the diagnostic criteria, the most frequently used now being the modified Duke criteria [1]. These challenges often lead to delayed diagnosis and treatment.

Identifying the microorganism involved in producing infective endocarditis remains the key of specific and early treatment. Blood culture is fundamental, but in some cases, the result is negative, up to 31% [1,3,4,5]. The main causes of blood culture-negative infective endocarditis are previous antibiotic treatment (up to 50% of cases), fastidious or facultative intracellular microorganisms, fungi or noninfectious endocarditis (Libman–Sacks, marantic, from lupus or Behcet disease) [3,6,7].

There is a need for useful biomarkers for early risk stratification, due to the poor prognosis of IE, more so in that with negative blood cultures. The absence of one specific parameter for predicting the disease’s course reflects the heterogenous physiopathology of infective endocarditis [8].

Renal and liver dysfunction usually develop during infective endocarditis, due to combined mechanisms. The Model for End-Stage Liver Disease Excluding International Normalized Ratio (MELD-XI) score is a composite between serum creatinine and bilirubin, ruling out the Internationalized Nominalized Ratio (INR)—which induces a false elevation in patients with anticoagulant treatment in the absence of liver dysfunction [9,10,11]. Nevertheless, the prior combined evaluation of hepatic and renal function could correlate with infective endocarditis risk profile and allow the early and intensive optimization of treatment, and a better outcome.

This study aimed to describe the epidemiological, clinical and paraclinical parameters in infective endocarditis to establish possible differences between blood culture-negative and blood culture-positive groups, and to explore the laboratory findings that are associated with in-hospital mortality.

## 2. Results

### 2.1. Baseline Patient Characteristics

A total of 126 IE patients were included in the final analysis (77% males). All the patients met the modified Duke criteria for “definite” and “possible” infective endocarditis and were >18 years old. The mean age at the time of diagnosis was 56.28 ± 15.35 years.

In the present study, 47.6% of the patients had a history of cardiovascular disease (CVD) predisposing for developing IE, and 17.5% had diabetes mellitus. The most frequent cardiac conditions that found to be predisposing to IE were mechanic or biological valvular prothesis (13.5%), aortic valve bicuspidy (7.1%), mitral valve prolapse (7.9%), congenital heart disease (3.9%) and history of IE (3.1%).

### 2.2. Blood Culture Results

Blood culture evaluation on admission showed negative results in 54% of the cases. The most frequent microorganisms detected in the blood culture-positive group were as follows: viridans streptococci (13.6%), *Enterococcus faecalis* (10.3%), *Staphylococcus aureus* (8.7%) and coagulase-negative staphylococci (4%). Before current hospitalization antibiotic treatment was documented in 20.6% of the total study group. The use of molecular diagnostic methods (polymerase chain reaction—PCR) was not available as a supplementary tool for revealing the microorganism for the study period in our center.

The study population was divided into the blood culture-negative IE group (*n* = 68) and blood culture-positive IE (*n* = 58) group, based on the blood culture results at the time of admission. The baseline characteristics of the two groups of patients are summarized in Table 1.

### 2.3. Laboratory Findings

The laboratory characteristics of the two groups are summarized in Table 2. The evaluation of biological parameters was as follows: C-reactive protein (CRP), erythrocyte sedimentation rate (ESR), fibrinogen, white blood cell count (WBC), with the major types of count (neutrophils and lymphocytes), hemoglobin level, platelet count, mean platelet volume (MPV), red cell distribution width (RDW), serum creatinine, estimated glomerular filtration rate (eGFR), bilirubin, serum proteins and MELD-XI score revealed different correlations with the blood culture result and disease outcome.

### 2.4. Echocardiographic Findings

A transthoracic echocardiography was performed for all the cases during admission. On the other hand, transesophageal echocardiography was performed in 33.3% of the cases, and valvular complications (abscess, chordae rupture, perforations and prothesis dehiscence) were revealed in 11.1% of the total cases, most frequently in the blood culture-positive group (7.1% vs. 4%; *p* = 0.121) (Table 1).

Concerning cardiac involvement, aortic valve endocarditis was most frequently observed (53.2%), followed by mitral valve (27%), prosthetic valve endocarditis (7.14%), bi-valvular endocarditis (6.33%), tricuspid valve (3.96%), pulmonary valve (1.58%) and pacemaker lead endocarditis (0.79%).

### 2.5. Outcomes and In-Hospital Mortality

The in-hospital mortality was 28.6% (*n* = 36) higher in male patients (77.8% vs. 22.2%). The antibiotic treatment was primary initiated empirical, with further modification based on the results of the antibiogram. The main associations of antibiotics used were penicillin + gentamicin, vancomycin + gentamicin, ampicillin + gentamicin, rifampicin + gentamicin, ciprofloxacin + ceftriaxone. Furthermore, an infectious disease physician adapted progressively the antibiotic treatment.

Diabetes mellitus did not present a statistically significant correlation with in-hospital mortality. We observed an increased trend for the prevalence of disease and in-hospital mortality for infective endocarditis over the 11 years of analysis.

Classic diagnostic for IE is represented by Duke modified criteria, that in 2015 were adapted and improved by the ESC guidelines (1). In 2016 and 2017, we noticed an increased number of IE hospitalized in our center (29 cases, 23.0%), compared to 2007 and 2008 (17 cases, 13.4%).

### 2.6. Predictors of In-Hospital Mortality

Receiver–operator characteristic (ROC) curve analysis determined the value of several laboratory tests in predicting in-hospital death (Table 3).

The best predictors for in-hospital mortality based on the ROC curve analysis and AUC results were neutrophil count (AUC = 0.824), WBC count (AUC = 0.724) and MELD-XI score (AUC = 0.700). Additionally, good predictors (AUC > 0.600) were as follows: RDW (AUC = 0.653), platelets (AUC = 0.629), CRP (AUC = 0.616), serum creatinine (AUC = 0.674), eGFR (AUC = 0.650) and bilirubin (AUC = 0.657).

## 3. Discussion

In this single-center observational study, we found that a significantly high percentage (54%) of the cases had negative blood cultures on admission. No major baseline differences were identified between blood culture-negative and -positive groups. Classic laboratory tests and new risk scores proved to have a good predictive value for in-hospital mortality.

Infective endocarditis was more commonly identified in male patients. The gender-ratio was the same as that reported in other studies [1,3,4]. The mean age at diagnosis was similar to that reported in the ESC-EORP European Endocarditis Registry (EURO-ENDO) [4] and the International Collaboration on Endocarditis Prospective Cohort Study (ICE-PCS) [12].

An important difference compared to other studies is the significant percentage of blood culture-negative IE—54% of the study group. Other studies reported lower percentages of blood culture-negative IE: ESC Guidelines <30% [1]; EURO-ENDO 21% [4]; ICE-PCS only 10% [12]; a study conducted by Duke University mentions a 12% incidence [13].

Previous antibiotic therapy, before blood culture determination, is reported as the major cause of negative results in IE patients [1,12]. Our study revealed a lower percentage of subjects in this category (20.6%), compared to ESC statistics. These results are probably due to a lack of data in our analysis related to information concerning pre-hospitalization antibiotic treatment, and we do not interpret this as a change in the spectrum of microorganisms.

Other possible causes of negative blood culture upon admission could be related to the sample collection, the volume of blood, the system and the medium of culture used. To decrease the probability of negative blood culture, the protocol implemented in hospital was strictly respected. The standardized system used helps in identifying possible bacterial determinants of IE. Nevertheless, several other causes can be responsible for negative blood cultures. Noninfectious endocarditis causes were excluded from the current study. Rare determinants, such as fungi, *Coxiella burnetti* or fastidious microorganism, scould be a cause of negative results. When suspected, specific mediums of culture should be used, and clinical suspicion should be indicated to the laboratory with the prolongation of the needed culture time. A slow growth and the lack of a specific medium for culture could be responsible for negative results. Serological tests for *Coxiella burnetii* can be used when this etiology is suspected. Even more rare determinants for negative blood cultures can be *Bartonella* spp., *Tropheryma whipplei*, *Mycoplasma* spp., *Brucella* spp. and *Legionella* spp. Specific tests or the use of biological molecular methods, especially multiplex PCR, can help in identifying the microorganisms responsible for negative blood cultures [1,14,15,16,17,18].

We did not identify major baseline differences between the blood culture-negative group and blood culture-positive group of IEs. This supports the connection between previous antibiotic treatment and a negative result of blood cultures, underlying the need for the supplementary use of diagnostic techniques to determine the microbiological substrate of infective endocarditis [14].

Our study revealed a classical distribution of microorganisms in the blood culture-positive group, with a still higher percentage of *Streptococcus* spp. and lower rate of *Staphylococcus aureus* involvement. We consider the lower range of *S. aureus* involvement in association with the reduced number of IE as being related to invasive procedures compared to other studies.

Aortic valve involvement was the most common site for infective endocarditis in our study, followed by the mitral valve. Other studies, on the other hand, have reported the mitral valve as the main site of disease [12]. We reported a lower range of prosthetic valve endocarditis (7.14%) compared to previous data: EURO-ENDO—30.1%; ICE-PCS—21%; Euro Heart Survey—26% [4,12,14].

Diabetes mellitus is a relevant comorbidity in infective endocarditis. Our study group had a relatively small percentage of diabetic patients, and we did not observe a correlation between diabetes mellitus and in-hospital death, compared to the literature data [1,13].

Valvular complications reported in our study (11.1%) had a similar range with those described in other studies and showed a significant association with blood culture-positive IE [12,19].

Several studies proved the predictive value of CRP for IE outcome, namely, in monitoring antimicrobial treatment. Its progressive decrease can suggest a favorable response, while persistent high values suggest complications and/or antibiotic resistance and induce the need for further investigations [20,21]. Our analysis also showed a good predictive value of high CRP levels for a negative outcome of disease.

This study found a good correlation between RDW and in-hospital mortality. RDW, an index of RBC heterogeneity, is used to assess long-term prognosis. RDW could prove useful in assessing the in-hospital and long-term outcomes of IE, a disease with complex physiopathology, including inflammatory and autoimmune mechanisms [22].

Another useful parameter, WBC count, correlated positively with a higher in-hospital mortality rate. Nevertheless, normal values may not predict a better outcome, as shown by other authors [21].

Thrombocytopenia proved to be a good biomarker in assessing the risk for a poor outcome in IE, similar to other studies [23,24]. This should not come as a surprise, given the determinant role of platelets in endothelial-related diseases.

Some of the biomarkers have a good correlation with blood culture results. ESR, neutrophilia and reduced levels of hemoglobin were significantly increased in patients with blood culture-positive IE. We considered this because of more virulent bacteria causing the infection, such as *S. aureus*.

Available studies showed increased MPV levels as an independent predictor of poor outcomes and high association with embolic events in IE. Contrary to it, our analysis did not reveal a correlation with in-hospital mortality for MPV. We consider this in the context of the reduced number of cases of IE with virulent microorganisms, such as *S. aureus*, which are associated with platelet dysfunction [25].

The MELD-XI score showed significantly higher values for blood culture-negative patients and proved to be a good predictor for IE outcome. By correlating it with the higher mortality for the blood culture-negative group, we can assume that the MELD-XI score is a good predictor of negative outcome to patients with blood culture-negative IE. We interpret this as consequences of extended and aggressive antibiotic treatment, with a wide antibacterial spectrum to cover all possible microorganisms involved in IE. Additionally, it is possible that microorganisms with slightly lower virulence are responsible for the blood culture-negative group, but with a longer disease course and progression until positive diagnosis and consequently more negative outcome.

We do not consider the ascending trend for diagnosis as a change of epidemiology of the disease, but the result of the improvement of diagnostic methods.

The in-hospital total mortality (28.6%) was similar to that reported by the ESC Guidelines [1], ICE-PCS [12] and several other studies [13], but higher than the recent EURO-ENDO report, placing it at 17.1% [4]. This finding indicates the dynamic nature of the disease without reduced mortality over time and a challenging disease with difficulties in diagnostic and treatment.

## 4. Materials and Methods

### 4.1. Study Design and Ethics

We conducted a retrospective observational study on infective endocarditis cases hospitalized in the Department of Cardiology of a regional hospital from North-Eastern Romania, from January 2007 to December 2017. The study protocol was approved by the ethics committee and the hospital review board. The investigation was in accordance with the Declaration of Helsinki.

### 4.2. Inclusion and Exclusion Criteria

Patients records were taken from the observation charts and from the electronic database and included baseline demographic data (age, sex and population distribution), clinical diagnosis, echocardiographic findings (affected cardiac structure, left ventricle ejection fraction (LVEF) and perivalvular complications), blood culture and laboratory results (the white blood cells count, neutrophil count, lymphocyte count, hemoglobin, platelets, red cell distribution width (RDW), mean platelet volume (MPV), C-reactive protein (CRP), erythrocyte sedimentation rate (ESR), fibrinogen, serum creatinine, eGFR, bilirubin, and serum proteins), MELD-XI score, complications in the disease course and in-hospital all-cause mortality data. All the patients were diagnosed with infective endocarditis based on the modified Duke criteria and the ESC guidelines improvement from 2015 (1). We used the results of blood cultures, echocardiography, computed tomography and all the necessary laboratory samples for minor criteria detection. For the time period included in the current study, no ^18^F-FDG PET/CT or SPECT/CT were available, neither molecular diagnostic methods.

**Figure 1:**
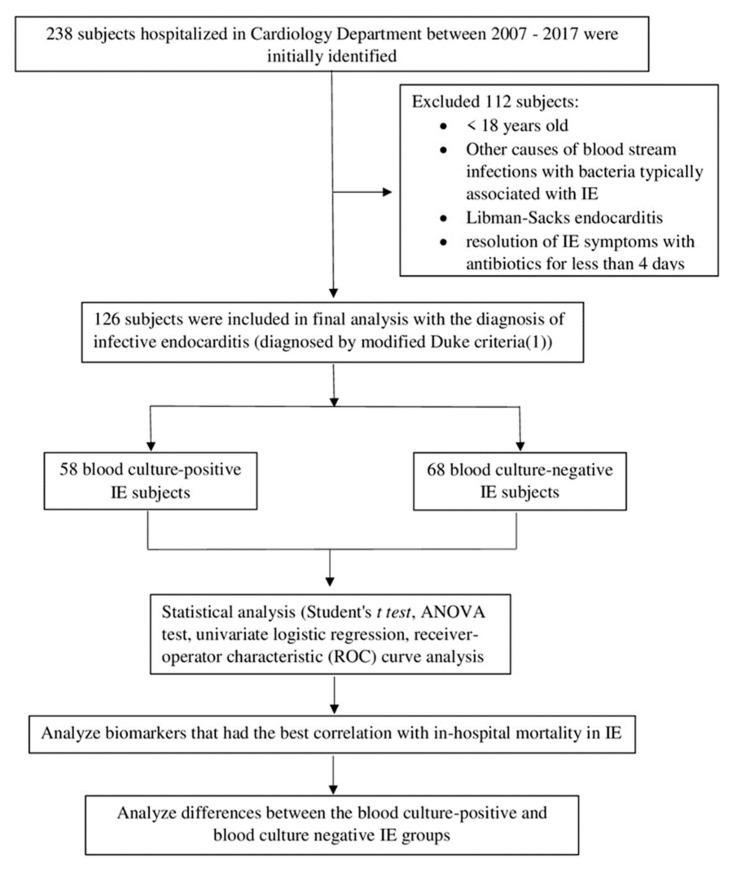
The flowchart of current study.

The total number of patients considering those excluded and the final numbers analyzed are outlined in Figure 1.

### 4.3. Blood Culture Determination

Before antibiotic therapy was initiated, an average of 3.17 sets of blood culture were obtained for the entire study group and an average of 3.82 sets for blood culture-negative IE. The system used for blood culture determination was Becton Dickinson, Le Pont de Claix, France (BD Bactec™, Becton, Dickinson and Company, Franklin Lakes, NJ, USA), with one bottle BD BACTEC™ Plus Anaerobic medium and one BD BACTEC™ Plus Aerobic medium. When there was a high clinical probability for fungal etiology, a fungi bottle was also used. The volume of blood collected for each determination was that indicated on each bottle (8–10 mL). The total volume of one bottle, after blood was collected and added to the culture medium, was 40 mL. Additionally, the laboratory protocol indicates “conform probs”, and if the volume is not respected as indicated by the protocol, the probe is considered inappropriate, and the blood sample was repeated.

### 4.4. Variables and Clinical Endpoints

We reviewed the medical records and screened for potential predictors, including routine blood markers, such as the complete blood count (CBC), inflammation, and liver and kidney function. We calculated the MELD-XI score [9] and observed the echocardiographic findings. The primary endpoint was in-hospital mortality.

### 4.5. Statistical Analysis

Continuous variables are expressed by mean ± standard deviation. The ANOVA test evaluates the characteristics of the study group. A Student’s *t test* was used to compare the differences between the two continuous variables. Categoric variables are represented as percentages and were compared with Pearson’s χ^2^ test. Receiver–operator characteristic (ROC) curve analysis was used to determine the value of several laboratory tests in predicting in-hospital death, based on the area under the curve (AUC). Statistical significance was defined by a *p* < 0.05. Statistical analysis was performed using SPSS version 18.0 software (SPPS Inc., Chicago, IL, USA).

### 4.6. Limitations of the Study

This was an observational retrospective cohort study and had methodological limitations. The information was retrieved from an electronic database and patient observation charts, which could lead to missing data, especially for antibiotic treatment prior to admission. The relatively small study group and the wide distribution over the 11 years of observation may have influenced the results, through limited statistical power and an era effect, respectively. In addition, no long-term outcomes were available.

## 5. Conclusions

Classic laboratory tests, such as white blood cell count and neutrophil count tests, together with newer tools, such as MELD-XI score, were associated with in-hospital mortality in infective endocarditis. A highly significant number of infective endocarditis cases had negative blood cultures on admission, but no major differences between the blood culture-negative and blood culture-positive groups were identified.

## Figures and Tables

**Table 1:** Baseline characteristics and echocardiographic features.

Variable	Blood Culture-Positive IE (58 patients)	Blood Culture-Negative IE (68 patients)	*p* Value
Age (years)	58.29 ± 14.68	54.55 ± 15.58	0.172
Male sex	45 (77.58%)	52 (76.47%)	0.477
Symptoms on admission Fever Dyspnea	47 (37.3%) 24 (19.0%)	46 (36.5%) 21 (16.7%)	0.066 0.149
Diabetes mellitus	10 (7.9%)	12 (9.5%)	0.571
CVD predisposing to IE	27 (21.4%)	33 (26.2%)	0.483
Previous antibiotic treatment	7 (5.6%)	19 (15.1%)	0.023
Affected cardiac structure Aortic valve Mitral valve Tricuspid valve Pulmonary valve ≥2 valves Prosthetic valve Pacemaker lead	26 (44.82%) 16 (27.58%) 5 (8.62%) − 3 (5.17%) 7 (12.06%) 1 (1.72%)	41 (60.29%) 18 (26.47%) − 2 (2.94%) 5 (7.35%) 2 (2.94%) −	0.560 0.067 0.732 − − − − −
Mean LVEF (%)	51%	51.16%	0.972
Perivalvular complications (valvular abscess, perforations)	9(7.1%)	5(4%)	0.121
Hospitalization period (days)	22.97 ± 15.98	17.59 ± 14.89	0.001
In-hospital mortality	14 (11.1%)	22 (17.5%)	0.207

Abbreviations: IE = infective endocarditis, CVD = cardiovascular disease, LVEF = left ventricle ejection fraction.

**Table 2:** Laboratory findings at admission.

Variable	Blood Culture-Positive IE (58 Patients)	Blood Culture-Negative IE (68 Patients)	*p* Value
White blood cells (10^3^/mm^3^)	12.73 ± 7.24	11.80 ± 5.77	0.434
Neutrophils (%)	78.25 ± 10.49	73.35 ± 12.16	0.025
Lymphocytes (%)	13.27 ± 7.56	17.38 ± 9.56	0.012
Hemoglobin (g/L)	10.37 ± 1.95	11.10 ± 1.96	0.042
Platelet (10^6^/mm^3^)	2.35 ± 1.36	2.29 ± 1.01	0.797
RDW (%)	16.57 ± 2.90	16.56 ± 4.82	0.989
MPV (fL)	10.05 ± 1.75	10.17 ± 1.15	0.670
CRP (mg/dL)	9.93 ± 9.47	13.56 ± 5.05	0.068
ESR (mm/h)	53.05 ± 34.88	38.53 ± 34.86	0.025
Fibrinogen (mg/dL)	446.27 ± 125.49	431.37 ± 143.24	0.570
Serum creatinine (mg/dL)	1.18 ± 0.75	1.36 ± 0.79	0.162
eGFR* (mL/min/1.73 m^2^)	78.77 ± 28.35	70.91 ± 33.15	0.164
Bilirubin (mg/dL)	1.21 ± 1.30	1.34 ± 1.22	0.692
Serum proteins (g/dL)	6.86 ± 0.72	6.89 ± 0.85	0.149
MELD-XI score	8.11 ± 5.24	12.36 ± 6.36	0.004

Normal values of laboratory parameters and abbreviations: White blood cells (10^3^/mm^3^) = 4.0–10.0, Neutrophils (%) = 45.0–80.0, Lymphocytes (%) = 20.0–45.0, Hemoglobin (g/L) = 12.0–15.5, Platelet (10^6^/mm^3^) = 1.5–4.0, Red Cell Distribution Width (RDW)(%) = 10.0–15.5, Mean Platelet Volume (MPV)(fL) = 8.5–12.0, C-reactive protein (CRP)(mg/dL) = 0.0–0.50, Erythrocyte Sedimentation Rate (ESR)(mm/h) = 0.0–20, Fibrinogen (mg/dL) = 200–400, Serum creatinine (mg/dL) = 0.50–0.90, Estimated Glomerular Filtration Rate (eGFR)(*CKD-EPI formula) (mL/min/1.73 m^2^), Bilirubin (mg/dL) = 0.20–1.20, Serum proteins (g/dL) = 6.6–8.7.

**Table 3:** Receiver–operator characteristic analysis for predictors of in-hospital death.

Variable	AUC	95% CI
WBC	0.724	0.510–0.939
Neutrophils	0.824	0.650–0.999
Lymphocytes	0.229	0.056–0.401
Hemoglobin	0.306	0.112–0.500
Platelets	0.629	0.378–0.879
RDW	0.653	0.433–0.874
MPV	0.229	0.074–0.383
CRP	0.616	0.424–0.809
ESR	0.512	0.246–0.779
Fibrinogen	0.502	0.220–0.784
Serum creatinine	0.674	0.563–0.784
eGFR	0.650	0.535–0.765
Bilirubin	0.657	0.516–0.797
MELD-XI score	0.700	0.580–0.843

Abbreviations: WBC = white blood cell count, RDW = red cell distribution width, MPV = mean platelet volume, CRP = C-reactive protein, ESR = erythrocyte sedimentation rate, eGFR = estimated glomerular filtration rate, MELD-XI score = Model for End-Stage Liver Disease Excluding International Normalized Ratio.

## Data Availability

The data presented in this study are available on request from the corresponding author. The data are not publicly available due to privacy and ethical reasons.
